# Barriers and Facilitators for Exclusive Breastfeeding within the Health System and Public Policies from In-Depth Interviews to Primary Care Midwives in Tenerife (Canary Islands, Spain)

**DOI:** 10.3390/ijerph19010128

**Published:** 2021-12-23

**Authors:** Seila Llorente-Pulido, Estefanía Custodio, María Rosario López-Giménez, Laura Otero-García

**Affiliations:** 1 PhD Programme in Public Health and Epidemiology, Preventive Medicine and Public Health and Microbiology Department, Faculty of Medicine, Universidad Autónoma de Madrid, 28029 Madrid, Spain; 2 Servicio Canario de Salud, Gerencia de Atención Primaria de Tenerife, Primary Health Care San Isidro, 38611 Tenerife, Spain; 3 CIBER Infectious Diseases (ISCIII), 28029 Madrid, Spain; ecustodio@isciii.es; 4 National Centre for Tropical Medicine, Health Institute Carlos III, 28029 Madrid, Spain; 5 Preventive Medicine and Public Health and Microbiology Department, Faculty of Medicine, Universidad Autónoma de Madrid, 28029 Madrid, Spain; mrosario.lopez@uam.es; 6 Nursing Department, Faculty of Medicine, Universidad Autónoma de Madrid, 28029 Madrid, Spain; 7 CIBER Epidemiology and Public Health (CIBERESP-ISCIII), 28029 Madrid, Spain; 8 National School of Public Health, Health Institute Carlos III, 28029 Madrid, Spain

**Keywords:** exclusive breastfeeding, qualitative research, midwife, health system, public policies, Spain

## Abstract

The aim of this study is to describe the perspectives of Primary Care midwives regarding factors that benefit or are detrimental to exclusive breastfeeding (EBF) within the health system and public policies. The study was carried out in Tenerife (Canary Islands, Spain) and is based on qualitative methodology. Twenty in-depth interviews were carried out with midwives who work in Primary Care centres in Tenerife, using a content analysis approach. The transcript data were then encoded following an inductive approach. The factors, according to midwives, that affect EBF, with regard to the healthcare system, are related to training of healthcare professionals in breastfeeding and their support to women during pregnancy, childbirth and postnatal care. Regarding public policies, midwives believe the maternity leave periods in Spain, together with a lack of laws and social policies to protect EBF are detrimental. The findings from our study show that there is a need to boost training and the role of professionals in EBF and, at the same time, promote protective policies that foster equality, favouring, among other issues, the work-life balance.

## 1. Introduction

The World Health Organization (WHO) recommends exclusive breastfeeding (EBF) during the first 6 months of life, and it has set the Global Nutrition Target for increasing the rate of EBF in the first 6 months up to at least 50% [1]. With these criteria in mind, the worldwide rates of EBF are still low (43%) and unevenly distributed; with regions like South Asia showing rates above 50% while West Africa have rates below 30%. The data from Europe are insufficient to establish proportions at the regional level [2], and the most recent statistics in Spain show an EBF rate at 6 months as low as 28% [3].

Taking this into account, the WHO member countries have been urged to reach the target by applying the measures necessary at the policy and health systems levels, to protect and promote EBF [1]. These include the promotion of publicity around EBF, the regulation of breast milk substitutes advertising, enacting 6 months mandatory paid maternity leave as well as policies that encourage women to breastfeed in the workplace and in public, the promotion of the Baby Friendly Hospital Initiative (BFHI) in healthcare systems, and the investment in training and capacity-building in EBF protection, promotion and support [1]. 

With regard to legislation around breastfeeding (BF) and appropriate marketing of breast milk substitutes, the WHO/UNICEF published the International Code of Marketing of Breast-milk Substitutes (1981) with the aim of regulating unethical commercial and advertising practices, without imposing BF [4,5]. Legislation around this Code is insufficient in most countries, with the so-called rich countries having less legislation than the so-called poor countries. In Europe, only 3 out of 53 countries have legislation in line with this Code [6], and in Spain there are only two legal documents that address the issue [7,8]. 

In relation to the enactment of policies to protect BF, the Innocenti Declaration (1990) on the Protection, Promotion and Support of Breastfeeding urges governments around the world to adopt the necessary measures to achieve a “breastfeeding culture” [9]. In relation to this, the European Union proposed in 2019 specific measures to promote work-life balance, including paid parental leave for both parents for a minimum of 4 months (2 of them non-transferable) and flexible work arrangements, among others [10]. 

Currently there is variation in Europe between states with regard to the duration, transferability and remuneration of maternal, paternal and parental leave (both parents) [11]. Bulgaria has the longest maternity leave (58 weeks) and Germany has the lowest (14 weeks) [12]. In Spain, the current maternity leave, which has not changed in the last 32 years, lasts 16 weeks [13]. On the other hand, the duration of paternity leave has significantly increased in recent years, starting from 15 days in 2007 to the current 16 weeks in 2021 [14]. Therefore, maternity and paternity leaves are the same, but they are not transferable. On another note, Spain is the only European country that has a so-called “breastfeeding leave”. There are several alternatives to enjoy this leave. Thus, although this permission is called “breastfeeding leave” it is intended to allow both parents to care for the child, without specifically addressing the BF needs [14,15].

The BFHI is a strategy that aims to improve care practices in childbirth and BF in maternity wards [16]. In Europe, countries such as Slovenia have the highest percentages of baby-friendly hospitals (93%), while France and Portugal have the lowest (2%) [17,18]. In Spain, only 3.1% of all hospitals and maternity wards are accredited as BFHI [19]. Moreover, the lack of support from healthcare professionals and inadequate hospital practices have been identified as important factors related to the low EBF in Spain [20,21].

In the Spanish Health System, some of the main actors involved in the promotion and support of breastfeeding among women are midwives, who have the competencies to provide advice, support and promotion of BF [22]. In particular, it is the Primary Care midwives who continue the care and monitoring of women after their hospital stay. Thus, they have a unique perspective on the positive and negative factors influencing EBF in that critical period.

The factors that influence the establishment of EBF interact at different levels which constitute a complex reality. Previous work by the authors of this study showed the insights of Primary Care midwives in relation to the barriers and facilitators of EBF within the women’s biopsychosocial spheres [23]. This article aims to complete these results. The study uses a qualitative research approach, collecting data with in-depth interviews, an appropriate technique to capture in-depth, detailed and individual perspectives of complex realities [24].

There are few studies in Spain addressing EBF using a qualitative perspective, and those that do, are from a mother’s perspective [25,26,27]. To the best of our knowledge, this study is the only one aiming to address EBF from the point of view of midwives.

This is the reason why we consider this study to be especially relevant, since it aims to determine, from the point of view of Primary Care midwives, the barriers and facilitators of EBF within the Spanish health system and public policies, and more particularly in Tenerife (Canary Islands).

## 2. Materials and Methods

**Figure 1:**
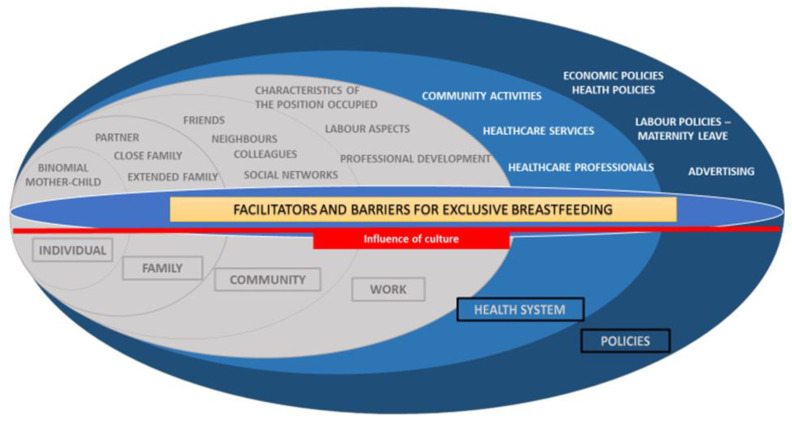
Barriers and facilitators for exclusive breastfeeding within the health system and public policies according to Primary Care midwives in Tenerife (Canary Islands, Spain). In the present article, we present the results related to the last two categories (in blue): health system and policies. Figure adapted from Llorente-Pulido et al. 2021 (in grey) and adaptation of Bronfenbrenner’s Ecological Model [23,28].

The materials and methods as well as the research conceptual framework presented here have already been published elsewhere [23]. The conceptual framework is represented in Figure 1 and is adapted from the Ecological Model of Bronfenbrenner [28], where the different levels that affect EBF are indicated, together with the results obtained per level. In a previous study we addressed the barriers and facilitators for exclusive breastfeeding (EBF) in women’s biopsychosocial sphere (individual, relationship, community and work levels) from the perspective of Primary Care midwives in Tenerife (in grey). In the present article, we present the results related to the last two categories (in blue): health system and policies. This second article completes all the different levels that affect EBF.

### 2.1. Study Design

The study was carried out on the island of Tenerife, belonging to the Canary Islands Autonomous Community in Spain. Tenerife is the largest of all the Canary Islands and the most populated of all Spanish Islands, due to increased birth rates and immigration. Regarding socioeconomic data, for the third quarter of 2021, the number of employed women in the Canary Islands was 406,400, corresponding to an employment and unemployment rate of 39.18 and 25.37%, respectively [29,30]. Considering the geographical definition of “Metropolitan Area/Zone”, Tenerife has 31 municipalities grouped into 11 counties, geographically located in the Metropolitan Zone, where its capital, Santa Cruz de Tenerife, is located, the North Zone and the South Zone. The population is greatly dispersed, which influences the organisation of health services. Most of the population live in urban areas (832,736 inhabitants), while 71,977 inhabitants live in a rural environment [29]. Tenerife’s health area has seven specialised healthcare centres and 39 Basic Health Zones (BHZ) with 101 healthcare centres, of which 39 are Health Centres (HC) and 62 are local practices [31] (Table 1).

The current number of Primary Care midwife staff in Tenerife is 53 (52 women and 1 man).

We performed a phenomenologically designed qualitative study with an inductive focus based on individual in-depth semi-structured interviews [24,32,33,34]. We recruited Primary Care midwives using a convenience snowball sampling technique. Initially, S.L.-P contacted the initial key informant via a phone message to their cell phone. This key informant is an experienced midwife in charge of coordinating all the BF groups on the island, and she referred her to the rest of the subjects. At this time, S.L.-P contacted the rest of the midwives in a similar manner, through a message to their cell phone, and they were successively interviewed until saturation was achieved (the point at which no new data were evident) in order to adequately represent the point of view of Primary Care midwives in Tenerife. During the interviews, an initial questionnaire was filled out to register characteristics such as age, workplace, type of population served, work experience, specific training in BF, whether they had children and whether their children were breastfed (see details in Appendix A), to develop different profiles. With the first eighteen interviews, saturation was achieved, but two additional interviews were conducted to fulfil the predefined set of profiles. Four midwives did not participate in the study due to lack of time to carry out the interviews.

A total of 20 in-depth interviews (20 out of the 53 Primary Care midwives of Tenerife) were carried out, 13 in the Metropolitan area, 3 in the North area and 4 in the South area. Among the interviewees there were midwives working for urban and rural populations, between the ages of 27 and 63 and with varied working expertise and BF training, as well as mixed motherhood experiences (Appendix A). 

The interview guide or script was developed by two of the authors, S.L.-P and L.O.-G. (PhD) and is included in the attachment (Appendix B). 

### 2.2. Data Collection

The study was presented by S.L.-P at one of the monthly meetings held by the Primary Care midwives in Tenerife. They use these meetings to present topics of common interest, update protocols and conduct continuous training courses. In that meeting, the objective, current state of the art, interest and reasons for carrying out the research study were explained.

The field work was carried out between the months of November 2018 and February 2020. The interviews were carried out by S.L.-P, who has a nursing degree with a specialisation in obstetrics and gynaecology (midwife) and is a fifth year PhD candidate, with qualitative research training, according to the availability of the midwives in the following manner: 3 in person, 1 by phone and 16 using a video call. These interviews took place either in their homes, workplace or in a cafeteria, and lasted between 30–60 min. In most cases, the interviewees were alone, except in three cases (where their children or students were also present). They were carried out a single time, without having to repeat any of them. All interviews were audio recorded and the interviewer collected field notes during the interviews, but no transcripts were returned to participants for comment and/or corrections. 

### 2.3. Ethical Considerations

The audio of the interviews was digitally recorded after receiving the participants′ written consent. Participants were informed about the objectives of the study and were guaranteed anonymity and confidentiality when expressing their opinions. They were assured that their participation was voluntary and that they could withdraw from the study at any time (Appendix C). 

This study obtained prior permission from the Tenerife Primary Care Management (Research Area) and approval of the Research Ethics Committee of the Autonomous University of Madrid (Madrid, Spain).

Throughout the article, we use the term “midwife” to refer to both women and men so as not to identify the speech made by the only man who is part of the sample.

### 2.4. Analysis

Interviews, carried out in Spanish, were transcribed verbatim by S.L.-P and an independent company. The first interview carried out was used as a pilot study. Data were anonymised prior to performing the analysis and participants′ names were removed from the transcripts and replaced by numbers. The transcripts of the interviews were analysed using a content analysis [35]. To facilitate the coding process, we (S.L.-P and L.O.-G) used the programme Open Code 3.6 [36]. Firstly, transcriptions were coded line for line, following an inductive approach that creates emerging codes that summarise the content of each sentence in a paragraph. The authors now provided a description of the coding tree for data analysis. Afterward, codes were categorised according to whether they were, in general, “facilitators” or “barriers” of EBF, to later identify them in a chronological order by subcategories (individual level of the woman, family, community, work, health system and public policies). The identification of topics was derived from the data. From the information obtained after the analysis, a framework was designed to structure the results (Figure 1). Participants did not provide any feedback on the findings.

## 3. Results

In this section we describe the results concerning the facilitators and barriers within the health system and public policies, following the conceptual framework structure. We provide participants´ quotations to illustrate themes but do not identify the specific participant providing it.

### 3.1. Health System

#### 3.1.1. Role of Community Activities in EBF

##### Aspects Regarding Community Activities That Hinder EBF

Lack of information about EBF in women who do not go to maternity/paternity preparation classes.

Midwives perceive that women who have not attended maternity/paternity preparation classes and have no contact with the world of BF at the family level, and do not have the initiative to document themselves, have more doubts and BF problems, often due to a lack of understanding of the dynamics and functioning of BF.

“*Those who have had problems with the latching are those who have not come to the maternity preparation classes and who have not learned the proper technique*.” (E15)

In the maternity/paternity preparation classes, women show greater interest about childbirth and less about BF.

Midwives agree that women who attend maternity/paternity preparation classes show greater interest in topics related to childbirth, while BF gets moved to the back burner. Midwives perceive that women show greater concerns and doubts regarding the actual moment of delivery. Therefore, they may not pay so much attention during the BF session and thus may not assimilate the information they are receiving, which causes a negative impact on the initiation of EBF and its adherence.

“*I have become convinced that women go to maternal education because the main issue is childbirth. Breastfeeding and the rest is not a priority*.” (E7)

##### Aspects Regarding Community Activities That Promote EBF

Participating in maternity/paternity preparation classes favours EBF.

Midwives highlight the importance of attending maternity/paternity preparation classes in order to acquire knowledge about the changes that occur during pregnancy, hygiene-dietary recommendations, information about labour and its phases, about BF, physical and psychological aspects during the postpartum stage and caring for the newborn. The objective of these classes is to allow women to be aware of these processes, normalise and express their feelings and offer them information that allows them to decide what is best for themselves and for their children. Specifically, in the BF session, its health benefits are explained, as well as how to carry it out both theoretically and practically, and what problems can be encountered, together with guidelines on how to solve them. All this is done with the main aim to motivate women to carry out EBF, by increasing their confidence and creating a climate of trust between the professional and the woman that will continue after the delivery, allowing problems and doubts to be adequately resolved at an early stage.

“*It is positive to talk about breastfeeding during the maternity classes, to give them some basic notions, and very clearly explain to them the things they have to be aware of, particularly regarding the breastfeeding technique.*” (E9)

Healthcare professionals need to approach BF in a simple way, to enhance learning.

The informants emphasise the importance of addressing BF in a simple way, without overloading the woman with too much information, so the message easily reaches mothers and fathers.

“*…give precise information, hassle-free… we should inform in the most natural way, not pressuring*.” (E16)

Attendance of couples to the maternity/paternity preparation classes to understand EBF.

Midwives perceive that more fathers are becoming involved in the processes of pregnancy, childbirth, BF and parenting, and they are increasingly more present in the maternity/paternity preparation classes. If couples are informed, they will better understand the dynamics of BF and, most importantly, the mother-child dyad.

“*If the couple has come to maternity education classes, if they have heard about breastfeeding, if they have understood what the milk production mechanism is like, if they are already clear on that, that´s a very important protective factor*.” (E5)

Establishment of support social networks for EBF during the maternity/paternity preparation classes.

Another objective of the maternity/paternity preparation classes, according to midwives, is the establishment of social networks and the creation of emotional bonds between future mothers and their partners as a present and future support network. This allows women not to feel alone during pregnancy and especially after childbirth, allowing these relationships to continue later when attending EBF workshops aimed at community health.

“*…people start coming to breastfeeding workshops when they have been in the maternity/paternity courses. There, a small women community is created, they interact with each other, a sort of bond is formed*.” (E4)

EBF workshops as a meeting point for mothers.

Midwives find the creation and attendance of EBF workshops to be a positive factor, enabling women to share their experiences and concerns, facilitating the resolution of many difficulties and breaking the myths surrounding EBF that midwives have identified.

“*…breastfeeding groups are a space where you can bring women together, you can observe all feedings and I think it also benefits them psychologically, because they see that many of their problems are shared*.” (E9)

Pregnant women attending EBF workshops to enhance motivation and self-confidence.

Midwives point out the importance of pregnant women also attending these EBF workshops so they can learn about the reality of postpartum and breastfeeding directly from other women. This allows pregnant women to be informed about EBF within a real context, where they can share doubts, and it also enhances the creation of women’s networks. The main objective of their participation in these workshops is motivation and empowerment of women.

“*…Women come who have been breastfeeding for two years, others who have been doing it for two months and others who have not given birth yet. So that encourages them a lot… I tell them: look, tell each other the problems you have had, how you started, what happened to you that you almost gave it up*.” (E11)

Allow couples and family members to attend the workshops to encourage support for EBF.

Midwives perceive a greater presence of partners and family members in maternity/paternity preparation classes than in EBF workshops. This could be due to the fact that in BF workshops there are other women who may expose their breasts which may make them feel uncomfortable. The informants point out the importance of including all the people around a nursing mother in the EBF process to promote their adherence and understanding.

“*One learns that the people around the woman are the mainstay of the person and you have to support and involve them. And I’m doing it and it works for me and it’s beautiful, it’s wonderful, it’s very beautiful*.” (E3)

#### 3.1.2. Influence of Healthcare Services in EBF

##### Hospital Practices that Impair the Early Onset of EBF

Lack of midwives in maternity wards negatively affects EBF.

The informants demand midwifery positions in maternity wards. Currently, postpartum wards are cared for by general nurses, without specific training to address postpartum and breastfeeding.

“*…They do not receive enough help from the postpartum wards, because sometimes they come across staff without lactation qualifications, because many wards are still occupied by general nurse practitioners and not by midwives” “… there is strong evidence that the midwife is the professional who has to be in a postpartum ward…*” (E9)

Contradictory messages from untrained personnel that make it difficult to start EBF early.

The presence of professionals untrained in BF during hospital admission negatively affects the early onset and adherence of women to EBF. If, during the first days, women receive contradictory and inadequate information about EBF, it can lead them to abandon it early.

“*…They always usually comment that, in the first days of being admitted, they can receive different information. They sometimes feel they don´t receive enough help or that they have even doubted things that were clear to them before and thus, miss out on exclusive breastfeeding*.” (E4)

Negative messages about EBF by healthcare professionals increase uncertainty among mothers.

This lack of training by professionals facilitates the spread of negative messages, creating uncertainty in mothers and hampering adherence to EBF and parenting.

“*…in the delivery room they are told to put the baby in the crib when they are alone in the room, not to do skin-to-skin contact. Those are the things that lead to insecure mothers*.” (E3)

Inadequate hospital practices around childbirth and caesarean sections make EBF harder.

Certain practices around childbirth and caesarean sections continue to exist that impede the early onset of EBF. There are evidence-based Clinical Practice Guidelines on care in normal childbirth and breastfeeding that encourage and promote the early onset of EBF during labour and after a caesarean section. Midwives recognise that positive changes are taking place in this respect, but a lot remains to be done.

“*The more complicated or perhaps more aggressive the delivery is, or less physiological, the more the establishment of breastfeeding is affected*.” (E9)

Indiscriminate supply of teats as a barrier to EBF.

The indiscriminate supply and introduction of teats, nipple shields, pacifiers or bottles during the hospital stay negatively interfere with EBF.

“*…many women go home from the hospital with nipple shields that later, when they come to the consultation, are very difficult to get rid of as the child has already gotten used to them…*” (E19)

Lack of an assessment of the baby´s feeding in the hospital, prior to discharge, hinders the establishment of EBF.

Midwives point out that an important barrier is the lack of assessment of BF prior to hospital discharge. This is a key moment for problems to arise. As soon as women get home, they may feel alone and incapable of identifying or solving problems.

“*…typical problems of cracks because there has not been a good assessment of the latching or problems of babies who are not suckling enough, a poorly established breastfeeding which has not been assessed*.” (E1)

##### Hospital Practices that Favour the Establishment of EBF

Midwives highlight that the current hospital practices, which follow the National Health System´s Strategy for normal childbirth care and the promotion of EBF, favour the early onset of EBF. The “Birth Plan” is a tool for the woman to indicate in writing how she would like the birth of her child to be and serves as a guide for professionals.

The Power of skin-to-skin contact to facilitate EBF.

Midwives emphasise that one of the practices that has changed the most and has facilitated the early onset of EBF is that, after birth, the newborn is placed in direct “skin-to-skin” contact with its mother. This practice, in addition to promoting EBF and providing warmth and protection, also creates a bond between mother and child.

“*The birth plans have been very beneficial; they have encouraged us to have skin-to-skin contact. There is evidence linking successful breastfeeding with skin-to-skin contact and early initiation of breastfeeding*.” (E3)

Ending the supply of bottles in maternity wards facilitates the implementation of EBF.

Midwives perceive that bottles are no longer offered indiscriminately in hospitals, enabling the newborn to learn the proper breastfeeding technique from the very beginning, thus not hampering EBF by the introduction of Artificial Feeding (AA).

“*…as the hospital no longer dispenses bottles so quickly, mothers try a little harder, as before they gave bottles all the time and now they give less…*” (E20)

Availability of EBF Information for the general public in the hospital.

Some midwives indicate that hospitals provide information that favours EBF and can be used to educate new mothers. This information is usually in visible places in the form of a poster or banners.

“*…I went to visit the delivery room, and on the baby carriages, in the cribs, there were little notes explaining how to interpret hunger cues*.” (E3)

#### 3.1.3. The Role of Healthcare Professionals in the Introduction and Maintenance of EBF

##### The Role of the Midwife in EBF

Characteristics of the care provided by primary care midwives which hamper EBF.

Non-protocolised postpartum consultations impair EBF follow-up.

Midwives recognise that pregnancy monitoring is much more rigorous and protocolised than it should be, unlike postpartum. The periodicity of postpartum appointments does not always occur at the right time, nor is there an established and consensual follow-up, but it often depends on the availability of the midwife. The postpartum check-up always takes place in a consultation office, and there is no home visit.

“*More support should be offered in the postpartum, because many times there is a stricter control in the pregnancy, once a month, and then in the postpartum the woman is left a little more neglected, with less control*.” (E9)

Lack of consultation time by Primary Care midwives to adequately see to BF problems.

The informants point out that the lack of time in consultation is one of the main problems in properly assessing BF. The care load negatively affects BF as, in order to adequately assess the baby´s feeding intake, time and patience are required, often for several days in a row. Very few midwives have specific consultation time dedicated to BF.

“*…because it is also important for women to be able to consult you, but sometimes you don’t devote all the time you really need, because your schedule is increasingly shortened and you cannot devote all the time the woman would need*.” (E14)

On the other hand, there are midwives who currently cover several Basic Health Zones (BHZ) during the week, being in different health centres throughout the week, and thus are not able to see the same women continuously. This undermines the support and advice regarding BF in their absence.

“*…not being able to be in the same consultation every day makes breastfeeding very difficult, because it is impossible to follow-up on any problem if you are only in a health centre two days a week*.” (E18)

Role of the primary care midwife as facilitator of EBF.

The midwife is considered a key person in order to promote EBF within the healthcare system.

Midwives highlight the importance of all professionals providing support to women throughout all processes, but particularly midwives, as they are considered to be crucial for promoting EBF.

“*Clinical practice guidelines recognise the midwife as the person in charge of coordinating all actions related to breastfeeding as well as the rest of the team…*” (E1)

The support and monitoring of the pregnancy by the midwife in Primary Care allows creating and maintaining a relationship of trust with the woman throughout the entire process, which favours the adherence of EBF.

“*It´s also positive, because in Primary Care you generally build confidence with the women and when they trust you, then many times they pay attention to your recommendations*.” (E9)

Dealing with EBF prior to delivery enhances maternal willingness to carry it out.

Midwives indicate that it is essential to address the issue of EBF prior to childbirth to inform women progressively, individually and with the adaptations necessary for each situation.

“*…before giving birth, we start weaving a web to start putting them in contact with the world of breastfeeding, as her interest at that point in time is not breastfeeding and we have to take that into account*.” (E7)

An early postpartum visit allows an early assessment of EBF.

Midwives emphasise the importance of having a postpartum visit in the first days, as it is the critical period to start EBF and subsequently maintain it.

“*From their last visit, I already programme their postpartum check-up appointment with them and I tell them: there we will talk about breastfeeding, I will see you as soon as you come back from the hospital, the next day, as there is when you see the problems of breastfeeding*.” (E10)

The midwife must respect the mother´s decision without blaming her.

The informants are aware that some women do not wish to breastfeed. The midwife must be informed of and accept each woman´s decision, respecting it and supporting them.

“*…us midwives need to clearly accept that there are women who chose not to breastfeed and this is also OK and we must work to not demonise them, we have to free women from that guilt …*” (E5)

##### Role of the Rest of the Professionals in the Primary Care Team in EBF

Healthcare attention by primary care doctors and nurses that hinder EBF (Table 2).

Primary Care health professionals, who are in continuous contact with women and their children once they come back to the health centre, play a key role in the establishment and maintenance of EBF. Lack of training in BF by family physicians, paediatricians, community nurses and paediatric nurses can lead to the cessation of BF.

##### Role of Primary Care Doctors and Nurses That Benefit EBF

On the contrary, when women come across professionals involved and trained in BF, they can be a fundamental pillar in maintaining EBF. It is very important for women to know which healthcare professional to go to in case of problems or doubts. As can be seen from Table 3.

### 3.2. Policies

#### 3.2.1. Specific Policies and Initiatives Surrounding EBF

##### Lack of Economic Policies to Support EBF

Little investment to promote and encourage EBF.

Midwives highlight the scarce investment in EBF by the public healthcare system, although they do recognise that a change is taking place and an effort is being made to improve this, though it is still limited.

“*I think that from above they are beginning to support projects now… but I believe that breastfeeding has never been supported, neither from above nor from the paediatrician, that is, I believe that breastfeeding doesn’t give money and things that don’t give money don’t have anyone to move them forward*”. (E8)

Professionals interested in BF are discouraged because the health and institutional system hinders EBF.

The informants indicate that many healthcare professionals are interested in BF, but they become discouraged due to the lack of institutional support to carry out activities or initiatives to promote it.

“*There are professionals who support breastfeeding and try to lead the way, but they are disappointed because if the leadership does not see the importance of breastfeeding or the broad benefits for the system and the whole of society, if they do not see it, they can’t give it any weight, so they also need training…*” (E3)

##### Economic Policies That Promote EBF

Updating healthcare policies to promote EBF.

Midwives acknowledge the existence of small changes in policies in favour of EBF by the National Health System, and specifically the Canary Islands Health Service.

“*…I think the Primary Care Management is doing very well because it is promoting breastfeeding and training of professionals in BF which is the basic pillar that affects us. Money is being invested in that…*” (E2)

Institutional initiatives that promote EBF.

There are initiatives that promote EBF, professionals are getting involved so this continues to advance and improve. Initiatives such as BFHI, launched by WHO and UNICEF to encourage hospitals and health services to adopt practices that protect, promote and support EBF from birth, are ongoing. Tenerife hospitals are working to obtain this certification. In addition, there are health professionals, who come together to carry out studies and activities that go in the same direction.

“*…I love the initiatives such as the one of a midwife from the Canaries University Hospital because they get to the newspapers, to the public opinion, “be a god mother to a first-time mother”, it’s a precious name that puts breastfeeding on the stage…*” (E3)

##### Labour Policies That Harm EBF

The duration of maternity leave is insufficient to cover EBF.

The informants point out that the duration of maternity leave is insufficient to cover EBF. In addition, these leaves, which would allow couples to organise themselves and prioritise when they are trying to decide how to maintain EBF, are not transferable.

“*…There aren’t many laws that really support breastfeeding. You have a maternity leave of 4 months, when they tell you to nurse your baby for 6 months…*” (E1)

Absence of leaves that would allow women to delay their return to work to maintain EBF.

Midwives refer to the scarce employment and legal possibilities available for women to delay the moment they return to work in order to maintain EBF for as long as possible.

“*What people are trying to do is join all the possible hours of breastfeeding together and return to work as late as possible just when the baby is already eating complementary foods.*” (E5)

##### Advertising as an Obstacle for EBF

Advertising around artificial feeding negatively affects EBF.

Midwives indicate that the advertising surrounding artificial feeding is a negative factor for EBF. Mothers are easy targets for the bombardment of advertising on artificial feeding, with a wide variety of products and accessories available. EBF does not generate money for many sectors, so its advertising is not profitable.

“*The pharmaceutical industry is very powerful. Perhaps this has a big influence on the promotion and prevention policies not being so strong, because if there is a pharmaceutical company behind with economic interests…*” (E9)

The ease of access to artificial feeding leads to abandoning EBF.

Midwives perceive the ease of access to artificial feeding and all its supplements as a barrier to EBF. Women acquire artificial feeding easily and, when faced with physical problems in the breasts, difficulties or situations in which the demand for milk by the newborn increases, the mother thinks her milk does not satiate her child or she is not producing enough milk, so she resorts to possible substitutes available to feed her child.

“*…easy access to artificial milk is negative for breastfeeding, because you go and it is no longer in the pharmacy, it is in the supermarket…*” (E6)

Advertising EBF in a way that is not real harms nursing mothers.

Midwives point to the fact that BF advertised in a non-real context harms nursing mothers, as they do not feel identified with, thus EBF adherence is impaired.

“*That image of a normal and active woman is missing, even if she´s breastfeeding.*” (E3)

##### Advertising in Favour of EBF

Advertising for the promotion of EBF facilitates adherence.

Midwives perceive a change in the advertising of EBF compared to years ago, although they are aware that it is not enough. Women who claim their role as nursing mothers in society are those most promoting this change.

“*Regarding advertising, it is true that in recent years there has been a lot of publicity in favour of breastfeeding. It can be seen in many media.*” (E9)

Greater visibility of EBF in society.

Midwives have observed that women are increasingly breastfeeding in public, making it more visible and normalised in society, thanks to the women who fight for it.

“*…there is an increasing presence of women breastfeeding on the street and in shopping malls, restaurants. I think society is accepting that part of breastfeeding.*” (E7)

**Figure 2:**
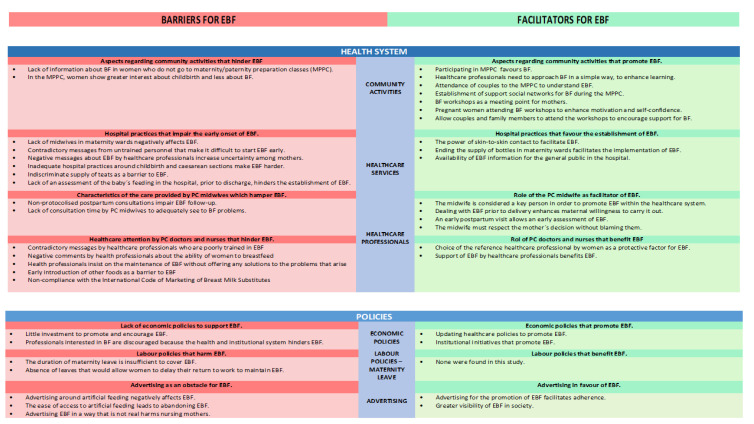
Facilitators and barriers for EBF within the health system and public policies according to Primary Care midwife´s perspectives in Tenerife (Canary Islands, Spain).

In Figure 2 we represent a summary of the results included in each section (Figure 2).

## 4. Discussion

Primary Care midwives of Tenerife present their perceptions about the different factors that positively and negatively affect EBF with regard to the healthcare system (taking into account community activities and health services and professionals) and different policies (economic, labour and health).

Within the health system, the barriers and facilitators for EBF were related to the care provided to women by health professionals during pregnancy, childbirth and postpartum. In relation to community activities (maternity and paternity preparation classes and breastfeeding workshops), the results of our study indicate the importance of future mothers attending classes prior to childbirth. The lack of adequate assistance to mothers, together with the consequent lack of information, is an important barrier to EBF. Midwives perceive that information given in prenatal classes on breastfeeding techniques facilitates EBF by preventing problems such as cracking and pain [37]. Other authors highlight the importance of women being informed as a method to prevent EBF cessation. The more informed the woman is, the more connected she will be with her pregnancy and her child, particularly regarding EBF [38]. On the other hand, midwives perceive that many women who do attend these maternity/paternity preparation classes seem to focus their concern on the time of delivery and the potential pain that accompanies it, leaving BF to a secondary position. This coincides with the attitude of women described in a study by Pascual et al. (2016) where women request that EBF be treated preferentially in postnatal classes, along with other topics such as care for the newborn and adaptation to motherhood [39].

For this reason, we emphasise the importance of receiving information on BF prior to childbirth, during the maternity/paternity preparation classes, and that all sessions are updated, well structured, and adapted to the working hours of future mothers and their partners in order to increase their attendance [39].

The midwives in our research also pointed out the importance of women’s partners participating in the sessions, since social networks of great importance for the initiation and maintenance of EBF are generated, as reflected in studies by Su et al. (2007), Ortega et al. (2010) and Pascual et al. (2016) [39,40,41]. Regarding postpartum EBF workshops, our results, similar to those found in other studies [21,42,43], suggest that midwives perceive that attendance at the workshops by women and their partners, or other members of their personal network, has a positive impact.

Regarding the time of birth, the midwives in our study identified that the barriers to EBF during the woman’s hospital admission are related to the lack of training of health professionals and the lack of a protocolised follow-up based on scientific evidence. This leads to practices that hinder early contact between mother and child as well as early onset of EBF, thus interfering with its establishment. Bandim Mariano et al. (2016) point out that the separation between mother and child after birth is associated with low levels of self-efficacy to carry out EBF, due to high levels of maternal stress and difficulties in understanding and identifying hunger signals in babies [44]. In addition, routine hospital practices, not based on scientific evidence, during vaginal delivery (inductions, episiotomies, unjustified obstetric manoeuvers) and caesarean sections (partner not present, no “skin-to-skin” contact with the mother and late onset of EBF), are detrimental to the onset of EBF, as reflected in the Clinical Practice Guidelines on care in normal childbirth and BF [45,46] and other studies [38,47,48,49]. The midwives interviewed highlighted that the lack of BF training of health professionals can lead to contradictory information being given to mothers (and their partners), by relating the anatomy of the breast with their ability to breastfeed, thus increasing their insecurity [50]. Another example of this lack of training is the indiscriminate supply of nipples (pacifiers, nipple shields or bottles) by health professionals in the first hours of the clinical postpartum period, which prevents the onset of EBF from being optimal [21,37]. Midwives reported that many women are discharged from hospital without a qualified health professional having evaluated the baby´s feeding intake. It is worth mentioning how important the assessment of the feeding intake is in order to intervene early and appropriately in each case [37]. The midwives interviewed expressed the need for all health professionals to be trained in BF, each one according to the role they play. Despite this, they claim that the figure of the midwife should not be limited to the delivery room in the hospital context, but that it is important that they can develop their care functions also in the maternity wards, accompanying women during other obstetric processes (pregnancy, postpartum and BF). The justification for this request is based on the fact that midwives have specific knowledge and skills for pregnancy, postpartum and BF care, as shown by scientific evidence [51,52,53]. Currently our healthcare system does not have legislation that can justify or regulate this request.

On the other hand, midwives also identify other hospital practices around birth that facilitate EBF, such as the practice of “skin-to-skin”, in accordance with studies by Gubler et al. (2013) and González et al. (2008) [38,42]. It is important that mother and child are not separated after delivery or a caesarean section [47,48,49]. All of this is included in the clinical practice guidelines on care in normal childbirth and BF; thus, following these recommendations is necessary for the optimal initiation of EBF, as shown in the study by Gutiérrez-Martínez et al. (2017) [47].

Regarding the postpartum period, the midwives in our study indicated that the absence of a protocolised postpartum visit once the mother and child return home from the hospital is a barrier to EBF. Their perception is that problems that may arise with EBF need to be addressed as early as possible. Women may feel lonely and need support for EBF-related problems in the first few days, so they should be cared for as soon as possible. The study by Rius et al. (2013) found that the time elapsed between hospital discharge and the postpartum follow-up by Primary Care is key for EBF [21], and Furnieles-Paterna et al. (2011) highlight that the ideal postpartum visit should be carried out by the midwife, in the mother´s house, within the first 48 h after discharge from the hospital [54]. The midwives in our study carry out the postpartum visit in the health centres, in the first days after delivery, depending on the availability of appointments (according to the available human resources). The duration of the check-up is approximately 20–30 min, which does not comply with an ideal postpartum follow-up visit [54]. In addition, they point out that there is no specific time devoted to BF during the consultation. The role of midwives as facilitators of EBF in Primary Care is essential, as clearly noted by the interviewees in this study and confirmed by other authors [21,37,42,55,56,57,58] The lack of human resources in Primary Care, thus leading to lack of time for health professionals, implies that midwives must take extra time from their daily consultation to help women with EBF [39,59].

As for the rest of the Primary Care health team (paediatric and community nurses, family doctors and paediatricians), midwives indicated that the lack of their support and training constitutes a barrier to EBF [20,60,61]. Our study suggests that women abandon EBF due to problems that could have been avoided through good professional counselling [62]. Midwives indicated that the absence of training by Primary Care health professionals leads to a lack of unification of criteria when dealing with EBF. This leads to contradictory and negative messages regarding women and their breastfeeding. An example that represents this lack of training for health professionals occurs when they advise women to introduce formula milk and other foods early (before 6 months), which also breaches the Marketing Code of Breast Milk Substitutes [20]. On the other hand, with respect to Primary Care health professionals acting as facilitators, the results of our study show their important role in promoting EBF. It is essential that health professionals can be a person of reference for women, someone they can turn to should they need to. Oribe et al. (2015) describe paediatricians and midwives as key professionals in preventing EBF cessation [62].

A lack of sufficient labour and social policies, not enough legislation to protect EBF, as well as a lack of institutional support for health professionals who are motivated to promote EBF, are some of the barriers to EBF with regard to public policies.

Midwives emphasise, among the barriers to EBF within the different political and health institutions, the lack of their support at a national as well as local level. At the national level, health policies that focus on tackling diseases and their clinical prevention limit the prioritisation and investment of primary prevention and health promotion strategies [63]. Taking into account the results of our study, the lack of investment by institutions in measures to enhance EBF and the absence of laws to protect it, mean that its promotion and support remain exclusively in the hands of health professionals and their willingness to spend time on this matter. Training in BF depends on the will of health professionals themselves who are often not motivated to promote EBF due again to the lack of resources, time and support from the institutions themselves, as previously observed in other studies [59,63,64,65,66,67].

Regarding the lack of time and healthcare resources, it is worth mentioning the effect the 2008 economic crisis had on our healthcare system, causing cuts in its services [68]. More specifically, Primary Care was one of the services that suffered the most in this regard, something that has become apparent with the arrival of the COVID-19 pandemic [69]. On the other hand, it should be noted that Spain is one of the European countries with the lowest public spending on health, which does not coincide with its economic development. In fact, Spain is one of the countries with the lowest spending in all public services related to the welfare state [70].

On the other hand, some facilitators of EBF within economic and healthcare policies are some institutional initiatives fostering EBF that have begun to appear, such as the desire by some health centres to obtain the initiative for the humanisation of childbirth care and breastfeeding (IHAN) accreditation, and the intervention programme “be a god mother to a first-time mother” [71]. Rius et al. (2013) also point out that the updating of policies and institutional initiatives regarding EBF are also facilitating factors [21].

Regarding barriers related to labour and social policies, the results of our study show a lack of integration of the gender perspective. Despite the fact that the introduction of women into the labour market is a reality, and despite the existence of policies to promote equality, the truth is that in our context, certain social and family policies have not been developed sufficiently, such as the family care and support benefits, which currently overburden women [72,73,74]. Specifically, the midwives in our study indicate the insufficient duration of current maternal leave to cover the duration of EBF, coinciding with the results found in the study by Oribe et al. (2015) [62]. The BF leave was not mentioned by any of the midwives of the study, thus confirming the consideration that this leave does not specifically promote BF. A leave of one hour per day is not enough to maintain EBF, and the alternative cumulative BF leave only adds 21 days to the 16 weeks of parental leave, thus remaining insufficient to cover the 6 months EBF period recommended by the WHO. In the case of Sweden, maternal leave is longer (480 days), thus covering not only the duration of EBF, but also the rearing of the child in the subsequent months. In this sense, it is worth comparing the context of our study with others such as Norway. Not only is there a greater pro-breastfeeding awareness in Norway, but there are social and labour policies that facilitate family life, with greater health benefits associated with BF, and additionally the training of health professionals is promoted [21,73,75,76]. On the other hand, in our context, midwives have not identified any policy or labour measures to promote EBF.

Finally, with regard to the existence of policies that protect BF from an advertising point of view, the midwives in our study indicated that the absence of penalties for non-compliance with the International Code of Marketing of Breast Milk Substitutes is a barrier [77]. Midwives point to the negative aspect of advertising in favour of formula milk and its ease of access. The adverts concerning EBF do not correspond to reality. In the study by Díaz-Gómez et al. (2016) they indicate that in the campaigns to promote BF, although its benefits are highlighted, the disadvantages of formula milk are not pointed out, implying that the latter is normal or good [20]. Despite this, the results of our research found that the small increase in EBF advertising, largely thanks to mothers themselves who make their breastfeeding visible and normalise it on social networks, is a facilitating factor. Society should integrate BF into daily activities and women who breastfeed in public should not be singled out or rejected [20].

Therefore, our study contributes to improving knowledge about the factors that benefit or harm EBF. The analysis of our results has important implications in health professionals´ clinical practice towards nursing mothers and their children. Our research also has repercussions for our legislators to guide and promote the development of public policies protecting EBF. For this reason, we emphasise that it would be very enriching to carry out studies taking into account the perception of women themselves about the positive and negative factors that influence their decision to breastfeed. In this sense, it would also be interesting and would complete this study, if the point of view of the rest of Primary Care professionals, as well as those involved in the care of childbirth and the immediate puerperium in the hospital setting, was investigated.

### 4.1. Limitations of Our Study

The study design allows for a rich description of the complex reality of midwives’ perceptions on barriers and facilitators of exclusive breastfeeding in Tenerife, although it does not allow the establishment of a hierarchy in the results, that is to identify which are the most important factors or those that most affect EBF. The convenience sampling hinders the possibility to generalise the results of the survey to the population as a whole, but we consider that the snowball technique applied allowed us to saturate the discourse of the Primary Care midwives according to their different profiles.

On the other hand, the use of qualitative methodology does not allow the results obtained to be extrapolated to other populations. The health, labour and social policies, as well as pro-breastfeeding culture are heterogeneous and dependent on the context, thus limiting the generalisation of our conclusions across settings.

### 4.2. Strengths of Our Study

The results of our research are of great interest due to the scarcity of studies on the perception of Primary Care midwives of Tenerife on the barriers and facilitators for EBF and the limited bibliography about the impact of policies on EBF beyond the health sector overall. In addition, the use of qualitative methods provides an in-depth and detailed study on these perspectives which cannot be feasibly obtained using quantitative methods.

## 5. Conclusions

The findings from our study indicate that Tenerife´s Primary Care midwives highlight several barriers and facilitators of EBF related to the healthcare system and public policies, greatly influencing its adoption and maintenance. According to the midwives’ perspectives/experiences, these factors not only affect the decision of women to breastfeed, thus putting at stake the nourishment of the infant from a nutritional point of view, but also have an impact on health, parenting style and socio-emotional development during childhood. The lack of development of economic, labour and health policies protecting motherhood/fatherhood, upbringing and childhood, have a clear impact on the WHO objectives to be achieved regarding EBF. On the other hand, the austerity measures adopted after the 2008 economic crisis, which have not been reversed, have led to little investment, particularly in Primary Care in the last decade, where there are limited human and health resources. The COVID-19 pandemic has shown and accentuated this lack of human and material resources in Primary Care, which could be jeopardising the promotion and support of EBF.

EBF must be approached from a gender perspective, demystifying and liberating women from being solely responsible for its success or failure. In order to do this, EBF must be tackled at a higher level, with social and collective competencies, since it not only affects the health of babies and mothers, but also has an impact on the health of the family, the community, and, ultimately, the entire society. The health system must promote the training of health professionals (especially those who are in contact with women and their children) in BF, requiring such training as an essential part of their clinical practice. For this, it would be positive to adequately develop this theme in undergraduate teaching. On the other hand, it is very important that the International Code of Marketing of breast milk substitutes be enforced in all health centres. The results of our study highlight the need to develop policies to protect the mother-child binomial and the whole family. For this, the development of public policies that prioritise equality and the integration of a gender approach is essential.

## Figures and Tables

**Table 1:** Inhabitants according to the area of the island, taking into account foreign population (ISTAC 2018), employed population (ISTAC 2020) and BHZ (https://www3.gobiernodecanarias.org/sanidad/scs/mapa) (accessed on 26 May 2020).

	North	South	Metropolitan	Total
Number of inhabitants	224.047	291.706	388.960	904.713
Number of foreign inhabitants	14.218	81.875	21.103	117.196
Employed population (in thousands)	93.42	129.78	172.04	395.24
Number of BHZ	12	10	17	39

**Table 2:** Healthcare attention by Primary Care doctors and nurses that hinder EBF.

Subcategories	Quotes from the Midwives
**Contradictory messages by healthcare professionals who are poorly trained in EBF** The informants point out the lack of training of healthcare professionals as an important negative factor for EBF. Conflicting and erroneous messages given to women can have dire consequences for EBF.	*“…they do it with good will, but obviously when you are not up-to-date, then you talk about what you have gone through, what you remember was done ten years ago, and so you give wrong information which causes anxiety to the patient.”* (E12)
**Negative comments by health professionals about the ability of women to breastfeed** Midwives indicate that negative comments, without scientific evidence, made by health professionals about the anatomy and functionality of the breast can lead to the cessation of EBF, since they foster uncertainty in women.	*“Sometimes, it´s not so much about the wrong advice, but how I impose my advice. It´s best not to say anything than to say: ‘Oh, but you don’t have milk!’ ‘Oh, your breasts are soft, hasn´t your milk come in yet?’”* (E3)
**Health professionals insist on the maintenance of EBF without offering any solutions to the problems that arise** The informants point out that Primary Care healthcare professionals insist on women continuing with EBF, despite the pain, without offering any solutions. The possible explanation for this could be the lack of training in BF.	*“But if it’s hurting and you don’t give me any solution, I have a crack and you don’t know what to do with me, you just scold me to keep breastfeeding in pain, well, women just give up breastfeeding…”* (E4)
**Early introduction of other foods as a barrier to EBF** Midwives regret the early introduction of formula and complementary feeding. The lack of scientific evidence of some recommendations given by some health professionals calls EBF into question.	*“The early introduction of complementary feeding by paediatricians is a tremendous battleground, and in the health centres there are no common criteria regarding this.”* (E5)
**Non-compliance with the International Code of Marketing of Breast Milk Substitutes** The informants claim it is necessary to monitor and penalise the cases where there is non-compliance in order to protect BF.	*“… The agreement for commercialisation of breast milk substitutes is not complied with, it is almost always violated in all centres. The professionals themselves do not comply with it and there is no follow-up to see who complies and who does not, and there is no repercussion for those who do not comply…”* (E2)

**Table 3:** Role of Primary Care doctors and nurses that benefit EBF.

Subcategories	Quotes from the Midwives
**Choice of the reference healthcare professional by women as a protective factor for EBF.** Midwives highlight the importance of the woman having a reference healthcare professional for her EBF to be successful. They also point out that it is important for the entire Primary Care team who is in contact with the woman and her child to have adequate training and give the same information.	“*…it is important for women to have a reference, at the professional level, who encourages breastfeeding, has knowledge about breastfeeding and knows how to solve difficulties…breastfeeding usually works…”* (E1)
**Support of EBF by healthcare professionals benefits EBF.** Midwives consider the support of EBF by healthcare professionals essential to promote and maintain EBF. Women need to feel accompanied to solve the difficulties that may arise during EBF.	“*The most important thing is that they do not feel alone, that they don´t feel abandoned, that someone is listening to them, that pain is given the necessary importance even if it has no physiological or anatomical explanation…”* (E3)

**Figure A1:**
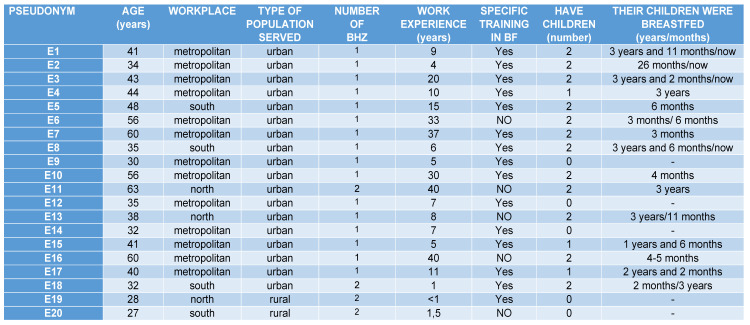
Profiles of Primary Care midwives.

## Appendix B. Interview Script for Midwives

### Appendix B.1. Facilities for Women to Breastfeed

Based on your experience in consultation, what facilitating aspects do women verbalise when breastfeeding?

Do they have support at the family level? Do they have support from their partner? Do they have the ability to continue breastfeeding while at work?

### Appendix B.2. Barriers or Obstacles Women Have to Breastfeed

Based on your experience in consultation, what barriers for breastfeeding do women verbalise?

What are the most common problems women encounter during the first days when breastfeeding?

What are the most common problems women encounter when breastfeeding once breastfeeding has been established?

At what point do they have the most difficulties: at the beginning or in the following months?

Do they have breastfeeding problems after returning to work? What do women do to reconcile breastfeeding and work?

Where do they more easily express their problems: in consultation or in breastfeeding workshops?

### Appendix B.3. Perceptions about the Causes of Breastfeeding Abandonment

In your experience, what factors do you consider responsible for the abandonment of breastfeeding during the first six months?

### Appendix B.4. Perceptions about the Causes of Breastfeeding Adherence

In your experience, what factors do you consider responsible for adherence to breastfeeding during the first six months?

### Appendix B.5. Proposal to Improve the Encouragement and Promotion of Adherence to Breastfeeding

Currently, what do you do in your consultation to promote breastfeeding adherence? What could you do and what resources would you need to improve your care in this regard?

What measures would you propose at the social level to encourage and promote breastfeeding?

What measures would you propose to the health system to encourage and promote adherence to breastfeeding?
